# Artificial intelligence-empowered echocardiography: an updated review of clinical management in hypertrophic cardiomyopathy

**DOI:** 10.3389/fcvm.2026.1787664

**Published:** 2026-08-27

**Authors:** Miao Zhang, Shanshan Yuan, Hongyan Dai, Guoan Wang

**Affiliations:** Department of Cardiology, Qingdao Hospital, University of Health and Rehabilitation Sciences (Qingdao Municipal Hospital), Qingdao, Shandong, China

**Keywords:** artificial intelligence, clinical management, deep learning, echocardiography, hypertrophic cardiomyopathy

## Abstract

Hypertrophic cardiomyopathy (HCM) is a common and highly heterogeneous inherited cardiomyopathy characterized by complex clinical phenotypes and diverse disease trajectories, posing significant challenges for early diagnosis, precise phenotypic classification, and risk stratification. Owing to its noninvasive nature, repeatability, and wide availability, echocardiography remains the cornerstone imaging modality for the diagnosis and longitudinal management of HCM. However, conventional echocardiographic analysis relies heavily on operator expertise and is limited in its ability to comprehensively extract latent structural, functional, and tissue-level information embedded within imaging data. In recent years, artificial intelligence (AI), particularly deep learning, has undergone rapid development in automated echocardiographic analysis, enabling a paradigm shift from traditional morphology-based assessment toward data-driven intelligent decision-support platforms. This review systematically categorizes AI-based methods in echocardiography according to the complexity of data processing, ranging from single-frame structural and texture analysis to spatiotemporal modeling of cardiac function, multi-view representation learning, and ultimately multimodal integration incorporating diverse clinical data sources. We further summarize the clinical applications of these AI-based methods in HCM, including diagnosis and differential diagnosis, phenotype characterization, and risk prediction. In addition, current challenges are discussed, including limited interpretability, data heterogeneity, and insufficient large-scale clinical validation, and future research directions are proposed. Overall, AI-empowered echocardiography holds substantial promise for advancing precision diagnosis, risk stratification, and personalized management of HCM, facilitating a transition toward more intelligent and individualized cardiovascular care.

## 1 Introduction

Hypertrophic cardiomyopathy (HCM) is a genetic cardiac disorder that is frequently underdiagnosed in clinical practice despite a relatively high prevalence of approximately 1 in 500 individuals (1). It is characterized by marked phenotypic heterogeneity and diverse clinical outcomes (2). The clinical presentation of HCM is highly diverse, ranging from long-term asymptomatic status to advanced heart failure (HF) and even sudden cardiac death (SCD) (3), posing substantial challenges for the development of individualized therapeutic strategies and long-term risk management. Consequently, achieving accurate early identification, dynamic assessment of disease progression, and optimized risk stratification has become a central issue in contemporary HCM clinical management.

Owing to its noninvasive nature, high reproducibility, broad applicability, low cost, and feasibility for bedside use, echocardiography has become the most widely used noninvasive imaging modality for the diagnosis, risk assessment, and follow-up of HCM (4, 5). However, conventional echocardiographic analysis relies heavily on operator expertise and is largely based on macroscopic morphological features and routine quantitative parameters, limiting its ability to comprehensively capture myocardial microstructural alterations and their underlying pathological substrates (5, 6). Previous studies have demonstrated that cardiac magnetic resonance (CMR) serves as an important complementary modality in left ventricular hypertrophy (LVH)-related diseases, including HCM, offering unique advantages in the precise assessment of hypertrophy distribution, myocardial tissue characteristics, and etiological clarification (6). Nevertheless, in real-world clinical practice, the widespread use of CMR is constrained by high costs, limited availability, and contraindications in certain patient populations. Therefore, extracting richer and clinically meaningful information from routine echocardiography without substantially increasing healthcare costs, to enable precise phenotypic characterization and early prediction of adverse outcomes in HCM, remains an urgent and unresolved clinical need.

In recent years, artificial intelligence (AI), particularly deep learning (DL) techniques, has achieved substantial progress in medical image analysis. Echocardiography, centered on two-dimensional grayscale dynamic imaging and supplemented by Doppler-based modalities, provides abundant information for DL-driven multimodal analysis. By enabling automated extraction and modeling of high-dimensional, complex features that are difficult to discern by the human eye, AI has introduced novel technical paradigms for the diagnosis and management of cardiovascular diseases (7–11), driving echocardiography to evolve from a traditional “visual assessment tool” into a “data-driven intelligent decision-support platform.” Emerging evidence indicates that AI-based echocardiographic analysis holds substantial promise in HCM diagnosis and differential diagnosis, phenotypic characterization, and risk prediction. However, comprehensive and systematic reviews summarizing methodological advances and clinical applications in this field remain limited.

**Figure 1:**
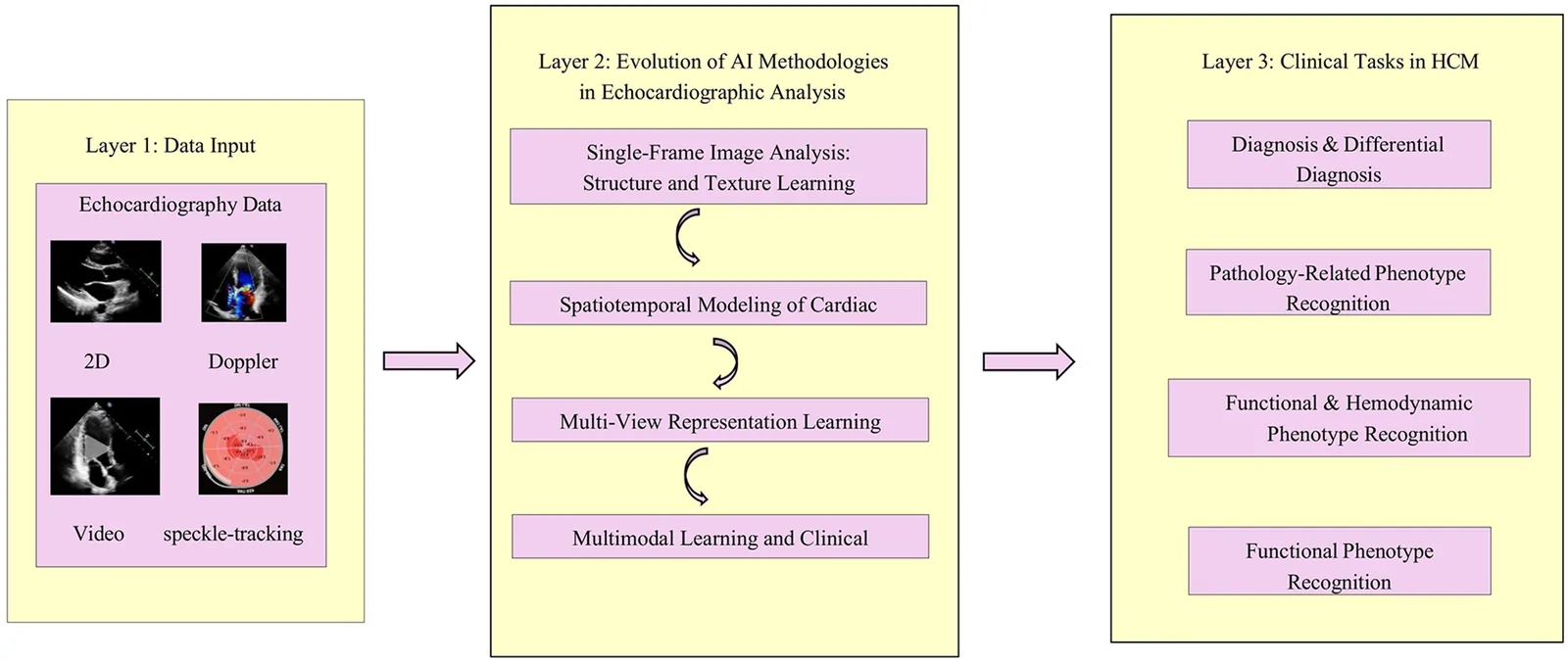
AI-empowered echocardiography in HCM: from data to clinical decision. AI, artificial intelligence; HCM, hypertrophic cardiomyopathy.

Against this background, the present review focuses on AI-empowered echocardiography. We first outline the core analytical methodologies and subsequently provide a systematic overview of recent advances in its application to HCM clinical management. Particular emphasis is placed on the value of imaging-based DL techniques in HCM diagnosis, phenotypic identification, and risk stratification (see Figure 1). Finally, we discuss current challenges and future directions, with the aim of informing clinical practice and guiding further research.

## 2 Research design and rationale

We conducted a scoping review to map the contemporary applications of AI-enabled echocardiography in the clinical management of HCM.

### 2.1 Methodological framework

This review adopted the five-stage methodological framework proposed by Arksey and O'Malley (12), which provides a structured approach to identifying, organizing, and summarizing existing evidence. Additionally, the PRISMA-ScR guidelines (13) were followed to ensure procedural transparency and reporting consistency:

1. clarifying the research question;
2. systematic literature search;
3. study selection;
4. data extraction and charting;
5. collating and reporting.

### 2.2 Research question

“What are the current research applications of AI-assisted echocardiography in the clinical management of HCM?”

### 2.3 Search strategy

As of May 2026, comprehensive searches were conducted sequentially in PubMed, Elsevier ScienceDirect, and Web of Science using the following query: (“artificial intelligence” OR “machine learning” OR “deep learning”) AND (“echocardiography” OR “cardiac ultrasound”) AND (“hypertrophic cardiomyopathy” OR “HCM”).

To maximize coverage, forward and backward citation tracking was performed to capture recent or yet-unindexed literature.

### 2.4 Inclusion criteria

Original studies (exploratory or validation studies) were included if they:

used AI methods;
enrolled HCM patients;
used echocardiographic images or echocardiographic measurements as input data;
targeted HCM clinical management, including diagnosis, differential diagnosis, phenotyping (pathological, functional, and hemodynamic phenotypes), and clinical risk prediction;
and reported quantitative performance metrics (AUC/ C statistics, sensitivity, specificity, accuracy).

Excluded were: review articles; case reports; records without full text; Artificial intelligence research employs electrocardiogram (ECG) or cardiac CMR, among others (rather than echocardiography), as the primary imaging modality; and preclinical/animal/computational simulation studies.

### 2.5 Study selection

The PubMed search yielded 74 records, of which 42 were excluded and 5 were added via citation tracking, resulting in 37 included studies. The Elsevier ScienceDirect search yielded 680 records (reviews and studies); after excluding those not meeting inclusion criteria and removing duplicates, 5 additional studies were added via citation tracking, all of which were included. The Web of Science search screened 136 full-text articles, of which 1 was ultimately included after exclusions. In total, 43 studies were included in this review.

## 3 Evolution of AI-based methods in echocardiographic analysis: from static imaging to multimodal intelligence

Early echocardiographic analysis primarily relied on handcrafted features and traditional machine learning (ML) approaches, such as random forests and active shape models (14, 15). With the rapid advancement of DL, convolutional neural networks (CNNs) have become the most widely used models in cardiovascular imaging-based DL research (16). Subsequently, temporal information across the cardiac cycle was incorporated through recurrent neural networks (RNNs) or temporal consistency constraints, enabling more stable and robust dynamic segmentation (17). In recent years, Transformer architectures and multimodal learning strategies have been introduced into myocardial analysis, facilitating the integration of imaging data with clinical information and laying the foundation for clinically deployable intelligent echocardiographic analysis systems (18, 19). A comparison of specific AI algorithms in echocardiography is presented in Table 1. This section organizes AI-based methods in echocardiographic analysis based on the increasing complexity of data.

**Table 1:** Comparison of AI algorithms in echocardiography.

Algorithm type	Representative methods	Typical tasks	Strengths	limitations	Current research status
Traditional ML	SVM, RF, k-NN, LR	Classification, risk prediction	Small data friendly; interpretable; fast	Needs manual features; limited complexity	Declining but useful in small datasets
CNN-based models	2D/3D CNN, U-Net	Segmentation, EF estimation, view classification	Strong spatial modeling; automated features	Weak temporal modeling; data sensitive	Most widely used and mature
Recurrent/Temporal models	LSTM, GRU, CNN-LSTM	Cardiac cycle analysis, motion tracking	Captures temporal patterns	Limited long-range dependency; complex training	Being replaced by Transformers
Transformer-based models	ViT, TimeSformer	Multi-view integration, video analysis	Global dependency modeling; flexible	High data & compute demand	Rapidly developing
Self-supervised learning	Contrastive learning, masked modeling	Pretraining, representation learning	Less annotation needed; strong transfer	Complex design; depends on fine-tuning	Key technique for foundation models
Foundation models	EchoJEPA	End-to-end multi-task analysis	Generalizable; multi-task; few-shot	Very high cost; limited interpretability	Frontier research area

AI, artificial intelligence; ML, machine learning; SVM, support vector machine; RF, random forest; KNN, k-nearest neighbors; LR, logistic regression; 2D/3D CNN, 2-dimensional/3-dimensional convolutional neural network; EF, ejection fraction; LSTM, long short-term memory; GRU, gated recurrent unit; ViT, vision transformer; TimeSformer, time-space transformer; EchoJEPA, echo joint embedding predictive architecture.

### 3.1 Single-frame image analysis: structure and texture learning

Early applications of AI in echocardiography were primarily based on static two-dimensional images, focusing on structural recognition and texture feature extraction. Structure-oriented approaches include view classification, chamber segmentation, and quantitative measurement of cardiac structures. Initial studies relied on handcrafted features and conventional ML algorithms (14), followed by the dominance of CNNs (20, 21), which enabled high-precision automated analysis and were subsequently extended to multi-structure and multi-view modeling frameworks.

In parallel, texture and tissue phenotype-oriented approaches aim to capture microstructural information embedded in grayscale images, facilitating the identification of disease phenotypes and underlying pathological states. Radiomics-based methods have demonstrated the discriminative value of texture features, while DL further enhances high-dimensional feature representation, improving performance in disease classification and prediction of pathological substrates such as myocardial fibrosis (MF) (22–24).

Overall, single-frame analysis marks the transition from subjective visual assessment to quantitative and automated analysis, providing a critical foundation for subsequent methodological advances.

### 3.2 Spatiotemporal modeling of cardiac function

Cardiac function is inherently dynamic and cannot be fully characterized by single-frame analysis. To address this limitation, spatiotemporal modeling incorporates information across the cardiac cycle to enable dynamic assessment of functional parameters.

Early approaches extended CNN-based frameworks by integrating RNNs or temporal consistency constraints, improving the stability of sequential analysis (25). Subsequently, video-based DL models, including three-dimensional CNNs and spatiotemporal convolutional networks, enabled end-to-end analysis of complete cardiac cycles and direct estimation of key functional indices such as ejection fraction (EF) (8, 26). More recently, Transformer-based architectures have been introduced to model long-range temporal dependencies through self-attention mechanisms, demonstrating advantages in capturing global cardiac dynamics (27, 28). Emerging approaches further integrate spatial and temporal features via attention mechanisms or prototype-based learning strategies, enhancing both predictive performance and interpretability (29, 30).

Spatiotemporal modeling represents a critical transition from static structural analysis to dynamic functional assessment.

### 3.3 Multi-view representation learning

Clinical echocardiographic assessment relies on multiple standard views, each providing complementary anatomical and functional information. However, early AI-based methods were predominantly developed using single-view inputs, limiting their clinical applicability.

Multi-view learning addresses this limitation by integrating information across different echocardiographic views to achieve more comprehensive cardiac representation (31–33). These approaches typically combine view-specific feature extraction with fusion strategies, such as attention mechanisms or multiple-instance learning, to aggregate cross-view information. This paradigm improves model robustness and captures spatial heterogeneity across imaging planes, more closely aligning with clinical practice, where diagnoses are based on the integration of multiple views.

Multi-view learning thus serves as a key intermediate step toward more advanced integrative modeling.

### 3.4 Multimodal learning and clinical integration

Building upon these advances, recent AI research has shifted toward multimodal learning and clinical decision support. These approaches integrate echocardiographic imaging with other data sources, including electrocardiography, CMR imaging, and electronic health records (EHRs), to improve diagnostic and prognostic performance (34–39).

From early incorporation of structured clinical variables to the development of vision-language models and multimodal Transformers (40), current frameworks enable joint encoding of imaging and textual data, supporting complex tasks such as automated report generation, disease classification, and risk prediction (41–43). In addition, multitask learning frameworks allow simultaneous execution of multiple diagnostic and measurement tasks within a unified model, enhancing efficiency (44).

Despite ongoing challenges, including data heterogeneity, limited interpretability, and insufficient large-scale clinical validation, multimodal learning represents a major future direction (38, 45), with the potential to transform echocardiography into an integrated clinical decision-support system.

## 4 From methods to disease: advances in AI-based echocardiography for HCM

In the preceding sections, we systematically summarized the core methodological pathways of AI in echocardiographic analysis. Ultimately, however, the true value of these approaches in medical imaging lies in their ability to improve diagnosis, phenotypic characterization, and risk stratification for specific clinical diseases.

Among cardiovascular disorders, HCM represents an ideal disease model for the validation and application of AI-based methods, owing to its marked phenotypic heterogeneity, challenges in early diagnosis, and substantial variability in clinical outcomes (46). AI-powered echocardiographic techniques are increasingly providing novel solutions for HCM diagnosis, phenotypic identification, and risk stratification.

### 4.1 AI for diagnosis and differential diagnosis of HCM

HCM exhibits highly heterogeneous phenotypes and often shows substantial overlap on echocardiography with other LVH-related conditions, such as hypertensive heart disease and cardiac amyloidosis. This overlap limits the early recognition and differential diagnosis of HCM when relying solely on conventional manual measurements and expert judgment. In recent years, AI—particularly ML and DL techniques—has emerged as a critical tool for automated identification and differential diagnosis of HCM based on echocardiography.

In 2016, Narula et al. (47) were the first to apply traditional ML to 2D speckle-tracking echocardiography, automatically extracting ventricular morphology and functional parameters. This approach provided quantitative metrics to differentiate HCM from athlete's physiological hypertrophy, marking the transition from handcrafted features to fully automated ML-based echocardiographic analysis and directly inspiring subsequent DL models.

To address the challenge of non-experts interpreting echocardiographic images, Zhang et al. (48), using a large-scale echocardiography dataset spanning ten years, demonstrated the feasibility of an end-to-end fully automated DL system in real clinical settings. This provided a key technical foundation for automated identification, precise quantification, and clinical decision support for HCM and other cardiac conditions.

**Figure 2:**
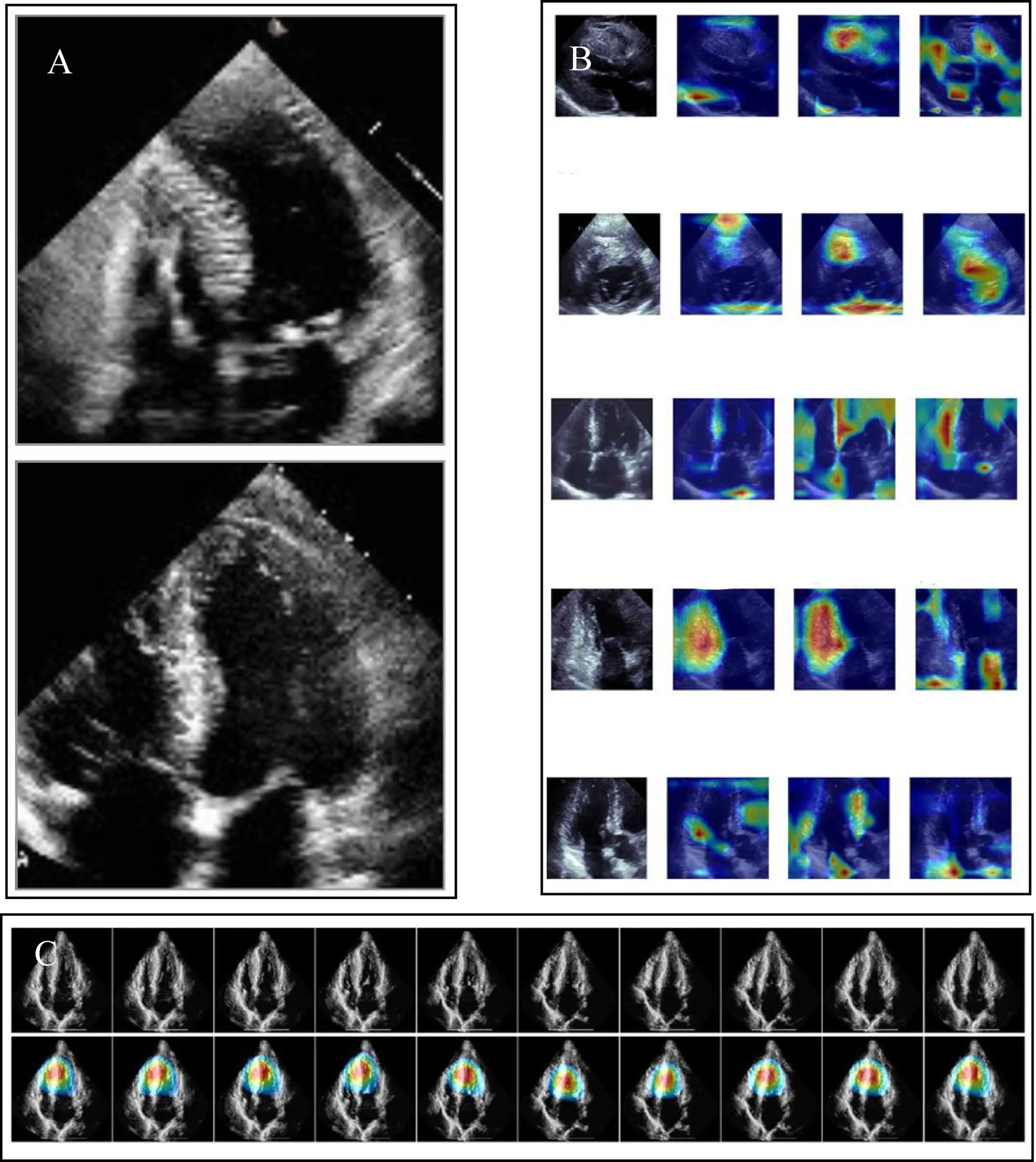
Visualizing machine learning models for hypertrophic cardiomyopathy diagnosis. Panels **(A–C)** display visualization outputs derived from machine learning models from different studies for the diagnosis of hypertrophic cardiomyopathy. Panels **(A–C)** correspond to references (49–51), respectively.

By 2022, studies specifically targeting differential diagnosis between HCM and other LVH-causing diseases began to proliferate. Duffy et al. (49) employed a multimodal DL approach to provide clinicians with an automated tool for LVH subtype classification, laying the groundwork for precise diagnosis of cardiac hypertrophy, including HCM. To address the “black-box” nature of DL, the study visualized model decision processes, revealing that highlighted regions were primarily concentrated in the interventricular septum, portions of the LV free wall, and areas showing abnormal cavity morphology or motion, consistent with typical anatomical and functional features of HCM (see Figure 2). In the same year, Hwang (50) and Liu et al. (51) validated DL-based approaches for classifying common LVH etiologies, also using visualization to emphasize the importance of septal regions in differentiating HCM from other causes of LVH (see Figure 2). To further enhance diagnostic performance, Shen et al. (52) proposed a multimodal diagnostic framework integrating echocardiography, ECG, and clinical data, enabling differential diagnosis of HCM and dilated cardiomyopathy, providing technical guidance for multimodal studies.

Additionally, to better identify genetic signature of HCM subtypes, Dai et al. (53) applied traditional ML to classify patients according to genetic features and clinical phenotypes, offering automated tools for personalized therapy, prognostic evaluation, and genetic research. Leveraging the portability of bedside echocardiography, Oikonomou et al. (54) employed DL methods to detect under-recognized cardiomyopathies, including HCM, thereby reducing missed diagnoses. Similarly, Farahani (55), Goto (56) and Vemulapalli (57) used ML tools to identify HCM patients.

Overall, a synthesis of existing studies (see Table 2) indicates that recent AI research has increasingly focused on the early and accurate identification of HCM and its differentiation from other LVH-related diseases, with multiple high-performing models emerging in this field (54, 58–66). The development of AI applications in echocardiography for HCM has followed a clear trajectory—from automated measurement and DL-based phenotyping to differential diagnosis and multimodal integration—providing strong support for precision diagnosis and early detection. Nevertheless, several challenges remain. Future research should focus on improving model interpretability, enhancing cross-center generalizability, promoting large-scale multimodal data integration, and conducting prospective clinical validation to facilitate real-world clinical translation.

**Table 2:** Summary of ML-based diagnostic and differential diagnosis methods and results for HCM.

Study	Input data	ML model	Study population	Validation type	Model performance
HCM diagnosis					
Zhang et al. (48)^a^ (2018)^b^	2D Echo videos (A4c, PLAX)	CNN	HCM	Internal validation	C statistics: 0.93 (HCM)
Goto et al. (56) (2022)	2D Echo videos (A4c, PLAX) + 12-lead ECG	3D CNN, federated learning	HCM vs. Non-HCM	federated learning	C statistics: 0.90–0.96 (HCM)
Dai et al. (53) (2023)	Clinical data, echo characteristics, and whole-exome sequencing data	Consensus clustering, DT, RF, LR + L1	HCM	External validation	NR
Farahani et al. (55) (2024)	Echo measurement indicators, demographic variables	RF	HCM	Internal validation	AUC: 0.98
					Sensitivity: 0.98
					Specificity: 0.83
Almadani et al. (58) (2025)	2D Echo videos (PLAX, PSAX, A2C, A4C)	DE (SlowFast + I3D + Majority Averaging Ensemble)	HCM vs. Non-HCM	Internal validation	AUC: 0.98
					Sensitivity: 0.94
					Specificity: 0.97
Vemulapalli et al. (57) (2025)	2D Echo videos (PLAX, PSAX, A4c, A2c, and A3c)	A CNN-based DL computer vision model [incorporating view classification and R (2 + 1) D encoder architecture]	HCM vs. Non-HCM	Internal validation	AUC: 0.85
HCM differential diagnosis					
HCM vs. ATH					
Narula et al. (47) (2016)	2D speckle-tracking Echo	SVM, RF, ANN	HCM vs. ATH	Internal validation	AUC:0.80
					Sensitivity: 0.96 (adjusted)
					Specificity: 0.77 (adjusted)
HCM vs. other diseases causing LVH					
Forghani et al. (59) (2021)	2D speckle-tracking Echo + ECG	SVM, KNN	HCM vs. HHD	Internal validation	AUC: 0.91–0.93
Duffy et al. (49) (2021)	2D Echo videos (PLAX, A4c)	CNN, Spatiotemporal CNN	HCM vs. CA vs. AS vs. other LVH	External validation	Internal dataset: AUC: 0.98 (HCM) AUC: 0.83 (CA)
					External dataset: AUC: 0.89 (HCM) AUC: 0.79 (CA)
Yu et al. (60) (2022)	2D Echo videos (PLAX and A4C)	CNN (ResNet, U-net++)	HCM vs. HHD vs. CA	Internal validation	AUC ≈ 0.92
Soto et al. (61) (2022)	2D Echo video (A4c) + ECG	Dual-branch multimodal DNN (1D-CNN for ECG + 3D/spatiotemporal CNN for Echo video)	HCM vs. HHD	Internal validation	AUC：0.91
Goto et al. (56) (2022)	2D Echo videos (A4c, PLAX) + 12-lead ECG	3D CNN, federated learning	HCM vs. HHD vs. AS vs. CA	Federated learning	C statistics:0.93 (HHD)
					C statistics:0.94 (AS)
					C statistics:0.85 (CA)
Xu et al. (62) (2022)	2D Echo videos (PLAX and A4C, Partially containing PSAX or A2c)	Video-level CNN, Spatiotemporal DL	HCM vs. HHD vs. UCM	Internal validation	AUC: 0.84 ± 0.049 (HHD vs. HCM)
					AUC: 0.87 ± 0.042 (HCM vs. UCM)
					AUC: 0.70 ± 0.140 (HHD vs. UCM)
Hwang et al. (50) (2022)	2D Echo videos (PLAX, PSAX, A4c, A2c, and A3c)	CNN-LSTM	HCM vs. HHD vs. AL CA	Internal validation	AUC: 0.98 (HCM)
					AUC: 0.96 (HHD)
					AUC: 0.10 (AL CA)
Li et al. (63) (2023)	2D Echo videos (PLAX, PSAX, A4c, A2c, and A3c)	End-to-end CNN	HCM vs. CA vs. HTN vs. others	Internal validation	fusion model AUC: 0.95 (HCM)
					AUC: 0.95 (CA)
					AUC: 0.92 (HTN)
Wu et al. (66) (2023)	Clinical data + conventional echo + speckle-tracking echo	LR, SVM, RF, GBM	HCM vs. AL CA	Internal validation	AUC: 0.98 (SVM)
Oikonomou et al. (54) (2025)	2D Portable Bedside echo Video (i.e., apical, PLAX, PSAX, and subcostal views)	3D-ResNet18	HCM vs. TTR CA vs. others (predominantly severe AS)	External validation	Internal dataset: AUC: 0.90 (HCM) AUC: 0.91 (TTR CA)
					External dataset: AUC: 0.89 (HCM) AUC: 0.85–0.97 (TTR CA)
Wang et al. (64) (2025)	2D Echo videos (A4c)	DTL	HCM vs. HHD + UCM	External validation	Training set AUC: 0.97
					Internal validation set AUC: 0.95
					External validation set AUC: 0.93
HCM vs. other common CVD					
Balaji et al. (65) (2016)	2D Echo videos (PSAX)	BPNN, SVM, KNN	HCM vs. DCM	Internal validation	Accuracy: 90.2
Liu et al. (51) (2023)	2D Echo videos (A4c)	Inception-V3, diagnostic NN (1D CNN + FC), CAM	HCM vs. DCM vs. ASD vs. MI	Internal validation	AUC: 99.57 (HCM)
					AUC: 99.50 (DCM)
					AUC: 98.75 (ASD)
					AUC: 98.52 (MI)
Shen et al. (52) (2023)	ECG images, Echo examination report data, and biochemical test data	CNN (ECG features), ANN, MLP	HCM vs. DCM	Internal validation	NR

ML, machine learning; CNN, convolutional neural network; DT, decision tree; RF, random forest; LR, logistic regression; L1, L1 regularization (Lasso); DE, deep ensemble; DL, deep learning; SVM, support vector machine; ANN, artificial neural network; KNN, K-nearest neighbors; DNN, deep neural network; CNN-LSTM, convolutional neural network—long short-term memory; GBM, gradient boosting machine; DTL, deep transfer learning; BPNN, back propagation neural network; NN, neural network; FC, fully connected; CAM, class activation mapping; MLP, multilayer perceptron; Echo, echocardiography; ECG, electrocardiogram; HCM, hypertrophic cardiomyopathy; ATH, physiological hypertrophy seen in athletes; LVH, left ventricular hypertrophy; HHD, hypertensive heart disease; CA, cardiac amyloidosis; AS, aortic stenosis; UCM, uremic cardiomyopathy; AL CA, light chain cardiac amyloidosis; HTN, hypertensive heart disease; TTR CA, transthyretin cardiac amyloidosis; DCM, dilated cardiomyopathy; ASD, atrial septal defect; MI, myocardial infarction; PLAX, parasternal long axis view; A4c, apical 4-chamber view; PSAX, parasternal short axis view; A2c, apical 2-chamber view; A3c, apical 3-chamber view; NR, not reported; AUC, area under the curve.

a Denotes the reference number.

b Denotes the publication year of the reference.

### 4.2 AI for pathology-related phenotype recognition in HCM

MF plays a central role in the pathological progression of HCM, directly leading to ventricular remodeling, diastolic dysfunction, and electrical instability, and serves as a key mediator of adverse clinical outcomes such as arrhythmias, HF, and SCD (67). As mentioned earlier, although echocardiography cannot directly visualize fibrotic tissue, its images contain rich texture, morphological, and dynamic information, providing opportunities for AI-based methods to uncover potential pathology-related phenotypes.

In recent years, researchers have attempted to leverage AI to extract fibrosis-related hidden information from conventional echocardiographic images. Liang et al. (68) applied a traditional ML approach based on echocardiographic radiomic features, constructing a classification model using myocardial texture features combined with the extreme gradient boosting algorithm to predict fibrosis-related phenotypes. Furthermore, Akita et al. (69) employed a deep CNN to analyze echocardiographic images and integrated it with a clinical reference model to develop a novel framework for predicting the presence of late gadolinium enhancement on CMR, an established imaging marker of MF. This model demonstrated superior diagnostic performance compared with conventional approaches, suggesting that DL can identify latent echocardiographic features associated with MF. In addition, ML-based extraction of echocardiographic texture features not only facilitates the identification of MF but also captures microstructural information that is difficult to assess using conventional echocardiographic measurements, thereby improving the differential diagnosis of HCM from other causes of LVH (22).

It should be noted that current AI-based methods based on echocardiography primarily achieve indirect identification of fibrosis-related phenotypes, rather than direct visualization of pathological changes. Nevertheless, as a noninvasive and low-cost screening tool, these approaches hold significant clinical potential, especially for patients who cannot undergo or are unsuitable for CMR imaging (68). In the future, with further integration of DL, temporal modeling, and speckle-tracking-derived multimodal features, echocardiography-based AI models are expected to play a greater role in identifying HCM pathological phenotypes and stratifying patient risk.

### 4.3 AI for functional or hemodynamic phenotype recognition in HCM

Functional abnormalities in HCM often appear before overt structural changes (70). Identifying these functional changes enables earlier risk stratification beyond morphological assessment. Early intervention can significantly improve prognosis in individuals at risk of HCM, transform current clinical practice, and pave the way for personalized management in cardiomyopathy.

To date, many studies have focused on integrating AI into echocardiography for precise measurement of functional indices in HCM. Global longitudinal strain (GLS) is used to demonstrate subclinical LV dysfunction in HCM, revealing impaired myocardial contractility even when EF is preserved (71). Zhang et al. (48) developed a CNN model capable of automatically measuring LS. More recently, Park et al. (72) developed the AI-based SMART system, which can automatically measure GLS. Additionally, Liu et al. (73) used a U-Net-based DL algorithm to automatically assess EF in HCM. However, Morrissey et al. (74) evaluated the performance of a commercially available automated LVEF assessment tool (Philips HeartModel®) in patients with HCM. Compared with standard expert transthoracic echocardiography interpretation and CMR, the automated tool systematically underestimated LVEF. These findings suggest that AI-based tools cannot directly replace expert interpretation and highlight the need for models specifically optimized for this patient population. In summary, DL methods now enable automated identification of HCM functional phenotypes, providing support for more precise therapy. The key challenge remains integrating these tools into clinical decision-making so that AI can serve as a robust instrument for HCM phenotype recognition.

LV outflow tract obstruction (LVOTO) is a central hemodynamic feature of HCM and directly influences the choice of therapeutic strategies and prognostic assessment (75, 76). In recent years, studies have attempted to apply AI to medical imaging for LVOTO evaluation. Sahota et al. (77) were the first to use linear and logistic regression to quantify the relationship between anatomical features and LVOTO in HCM patients based on CMR imaging data. Ning et al. (78) developed a multi-attention convolutional neural network (MANet) to identify high-risk echocardiographic features of HCM, including resting LVOTO, from 12-lead ECGs.

Echocardiography, as the primary tool for LVOTO assessment, has also become a focus of AI research. Park et al. (79) demonstrated that a DL approach could predict severe LVOTO using a single echocardiographic view, while Yuan et al. (80) developed an AI-based methods to detect LVOTO from apical four-chamber echocardiographic videos.

Systolic anterior motion (SAM) frequently coexists with dynamic LVOTO and serves as an important hemodynamic trigger for LVOTO (81). Echocardiography remains the preferred method for SAM diagnosis (81). Currently, no AI-based method is specifically designed for SAM detection, and clinical judgment remains the primary approach.

Overall, both LVOTO and SAM are highly dynamic and load-dependent hemodynamic phenomena whose severity may change substantially during exercise, the Valsalva maneuver, or other physiological provocations (82). Most currently available AI models are trained using resting echocardiographic images or short video clips obtained under stable loading conditions. Consequently, these models primarily learn structural and motion-related features present at rest and may fail to identify latent obstruction that becomes clinically evident only under altered physiological states.

In addition, conventional CNNs are optimized for spatial feature extraction and do not explicitly model changing hemodynamic conditions. Although video-based architectures, including recurrent neural networks and Transformer-based models, can capture temporal motion patterns, they generally rely solely on image sequences and lack direct information regarding preload, afterload, contractility, Doppler-derived flow velocities, and pressure gradients. Therefore, accurately predicting dynamic LVOTO severity and SAM-related obstruction remains challenging. Future advances will likely require multimodal AI frameworks that integrate echocardiographic videos with Doppler hemodynamic measurements, stress echocardiography data, and longitudinal clinical information to enable comprehensive assessment of complex LVOTO and SAM physiology.

### 4.4 AI for clinical risk prediction in HCM

Accurate prediction of adverse outcomes in HCM, including AF, SCD and HF, is essential for enabling early intervention and personalized management, thereby improving patient prognosis (83). In recent years, accumulating evidence has demonstrated the promising role of AI in risk stratification and prognostic assessment of HCM.

AF is the most common sustained arrhythmia in patients with HCM and is associated with an increased risk of stroke, HF, and mortality. Therefore, early identification of high-risk individuals is of great clinical importance. Bhattacharyya et al. (84) developed the HCM-AF risk model using ML techniques, integrating multiple clinical features, including echocardiographic parameters, to predict the risk of AF. Subsequently, Carrick et al. (85) established and externally validated the HCM-AF Score, which predicts incident AF using age at evaluation, age at HCM diagnosis, HF symptoms, and left atrial dimension measured by echocardiography. Rosca et al. (86) further developed a ML model incorporating clinical and echocardiographic variables to identify HCM patients with existing paroxysmal AF. More recently, Lu et al. (87) developed a ML model integrating multiple clinical variables and echocardiographic measurements, which demonstrated superior performance in predicting incident AF compared with the conventional HCM-AF Score.

Beyond AF, AI has also been applied to predict the risk of other arrhythmic events in patients with HCM. Bhattacharyya et al. (88) were the first to apply ML to HER data in HCM and developed the HCM-VAr risk model for identifying patients at high risk of ventricular arrhythmias. Wu et al. (89) pioneered the use of ML-based echocardiographic flow imaging to predict arrhythmic events in HCM. Furthermore, Lai et al. (35) developed a multimodal ML framework, MAARS, which integrates EHRs, echocardiography, radiology reports, and contrast-enhanced CMR data to predict life-threatening arrhythmic events in HCM, achieving superior predictive performance compared with current guideline-based risk stratification tools.

Progression to HF represents one of the most common adverse clinical outcomes in HCM. To address this challenge, Fahmy et al. (86) developed a ML-based prediction model to identify patients at high risk of progressing to advanced HF, thereby facilitating individualized disease management. In addition to HF, patients with HCM are also at risk of major adverse cardiovascular events (MACE), highlighting the importance of effective prognostic models. Kochav et al. (90) employed ML algorithms integrating clinical and echocardiographic-derived parameters to predict a composite endpoint of HF-related death, heart transplantation, and SCD. Rhee et al. (91) developed a ML model incorporating multiple phenotypic characteristics of HCM, which effectively identified patients at high risk of adverse cardiovascular outcomes, including all-cause mortality, HF hospitalization, and stroke. Notably, Wang et al. (92) were the first to apply DL and radiomics techniques to prognostic assessment using echocardiography in HCM. By automatically extracting high-dimensional imaging features from echocardiographic images, their model predicted MACE, including SCD, HF events, arrhythmic events, and AF-related stroke, thereby overcoming the limitations of conventional echocardiographic measurements and providing a novel strategy for AI-driven precision risk stratification.

Overall, substantial progress has been made in the application of AI for prognostic prediction in HCM. However, most existing models still primarily rely on clinical variables and conventional echocardiographic measurements. In contrast, studies directly utilizing echocardiographic images in combination with DL for predicting adverse outcomes remain limited. Future research should focus on exploiting the rich information embedded within echocardiographic imaging and developing image-based AI models to enable more accurate risk prediction and personalized management of patients with HCM.

## 5 Conclusion

With continuous advances in AI and iterative algorithm optimization, AI is establishing a comprehensive and efficient framework—from static imaging to multimodal analysis—delivering increasingly precise support for clinical practice.

In echocardiography, AI's superior data processing and image analysis capabilities are enhancing diagnostic and management performance. HCM exemplifies AI-assisted echocardiography: AI facilitates accurate and differential diagnosis, distinguishing features across LVH-related conditions, and enables deep phenotypic insights into morphological, functional, and hemodynamic characteristics. Moreover, AI supports risk stratification by providing robust, data-driven guidance for clinical decision-making. Taken together, AI-assisted echocardiography for HCM diagnosis and differential diagnosis has reached a relatively mature stage. Nevertheless, AI-based methods for phenotype recognition, particularly those addressing complex hemodynamic changes such as LVOTO and SAM, remain underdeveloped. Existing risk prediction models largely focus on arrhythmic events, with insufficient consideration of other adverse outcomes, including HF.

Although some progress has been made, challenges persist. Model “black-box” behavior, data heterogeneity, and limited generalizability constrain robustness and clinical reliability. Risk of bias remains an important concern in current AI studies of HCM. Many published investigations are retrospective and conducted at single centers, introducing potential patient-selection bias and limiting generalizability. Sample sizes are often relatively small compared with the complexity of deep learning models, increasing the risk of overfitting. Furthermore, imaging protocols, equipment vendors, and annotation standards vary substantially across institutions, contributing to data heterogeneity. Although internal validation is commonly performed, external validation cohorts remain limited, and prospective multicenter evaluations are uncommon. Consequently, reported performance metrics may overestimate real-world clinical effectiveness. Future studies should incorporate standardized methodological assessment frameworks, such as QUADAS-AI and PROBAST-AI, together with prospective multicenter validation strategies. Future AI development in HCM echocardiography is expected to emphasize visualization, multimodal integration, and multicenter collaboration. Visualization improves interpretability by highlighting clinically relevant structures; multimodal integration incorporates genomic and biomarker data for precise phenotyping and personalized risk prediction; and multicenter studies validate model generalizability across devices, protocols, and operators.

Systematically addressing these challenges will optimize HCM AI tools and accelerate their translation into reliable, broadly applicable clinical solutions. Furthermore, as AI matures, it is poised to enhance automated detection and risk assessment of LVOTO and SAM, advancing hemodynamic phenotyping. In the future, AI-driven echocardiography is expected to bring profound advancements to the clinical management of HCM.
